# Acute Urinary Retention Due to Incarcerated Uterus With Adenomyosis: A Case Report

**DOI:** 10.7759/cureus.63170

**Published:** 2024-06-25

**Authors:** Aalami Zeba, Khadija Ashraf, Kavitha Krishnan, Azhar Shabbir, Shanmugavel Chinnakaruppan

**Affiliations:** 1 Obstetrics and Gynaecology, Hamad General Hospital, Al Khor, QAT; 2 Obstetrics and Gynaecology, Hamad Medical Corporation, Al Khor, QAT; 3 General Surgery, Hamad Medical Corporation, Doha, QAT

**Keywords:** female bladder outlet obstruction, hysterectomy, acute urinary retention, uterine incarceration, adenomyosis

## Abstract

Acute urinary retention caused by uterine incarceration due to adenomyosis is a rare phenomenon with limited documented cases. This report details the case of a 38-year-old female with acute urinary retention. A pelvic MRI revealed an enlarged retroverted uterus with a mass on the posterior left of the myometrium, indicative of adenomyosis. The size and anatomical location of the mass suggested uterine incarceration with direct pressure on the urinary bladder neck, leading to urinary retention. The patient underwent total abdominal hysterectomy, bilateral salpingectomy, and adhesiolysis. Subsequent follow-ups at one and three months post-surgery showed the resolution of urinary symptoms, underscoring the importance of considering adenomyosis as a potential cause of urinary retention in women with lower urinary tract symptoms. Timely recognition and appropriate intervention are crucial for mitigating complications and improving outcomes in these patients, as illustrated in this case.

## Introduction

Adenomyosis of the uterus is characterized by the benign invasion of endometrial glands and stroma into the myometrium, leading to uterine enlargement and asymmetric thickening of the anterior and posterior myometrial walls [1,2]. Its prevalence is estimated to be between 20% and 35% globally [3-5]. Common presenting symptoms include abnormal uterine bleeding, pelvic pain, and infertility. While an enlarged uterus is occasionally known to exert pressure on the bladder, resulting in irritative symptoms such as nocturia and frequency, obstructive symptoms are typically uncommon. However, it is exceedingly rare for adenomyosis to cause such significant enlargement leading to uterine incarceration at the pelvic brim subsequently resulting in complete urinary obstruction. The postulated mechanism is direct pressure on the bladder neck or outlet, which may have contributary factors such as pelvic adhesions from previous surgery, pelvic inflammatory disease, or endometriosis. Uterine malformations, a gravid uterus, and the presence of fibroids increase the risk of uterine incarceration [6]. We present a case of acute urinary retention in a patient with adenomyosis to underscore the importance of considering it in the differential diagnosis of urinary retention in women.

## Case presentation

Our patient, a 38-year-old woman of Filipino ethnicity and a domestic helper by profession presented to the primary healthcare center with a one-day history of inability to pass urine and lower abdominal pain. She reported experiencing urinary frequency 8-10 times per day and a sensation of incomplete bladder emptying for the past three weeks. Her medical history included intermittent heavy menstrual bleeding and dysmenorrhea over the preceding three years, with no prior episodes of urinary tract infection, urinary retention, constipation, or neurological disorders. Upon initial assessment at the health center, she was hemodynamically stable with normal vital signs, and an abdominal examination revealed lower abdominal tenderness and mild distension. Bedside ultrasound of the abdomen demonstrated bladder distension, and subsequent catheterization yielded 1200 ml of urine. She was then referred to the obstetrics and gynecology emergency department for further evaluation and management.

Upon examination in the obstetrics and gynecology emergency department, her vital signs remained within normal limits. Abdominal examination revealed a soft, lax abdomen with mild suprapubic tenderness. Vaginal examination showed an enlarged retroverted uterus, equivalent to approximately 12 weeks in size, with the uterine cervix displaced anteriorly. Palpation revealed a mass in the posterior fornix, and the urinary catheter remained in situ. Positional and transvaginal manual maneuvering failed to mobilize the uterus.

Investigations

**Figure 1:**
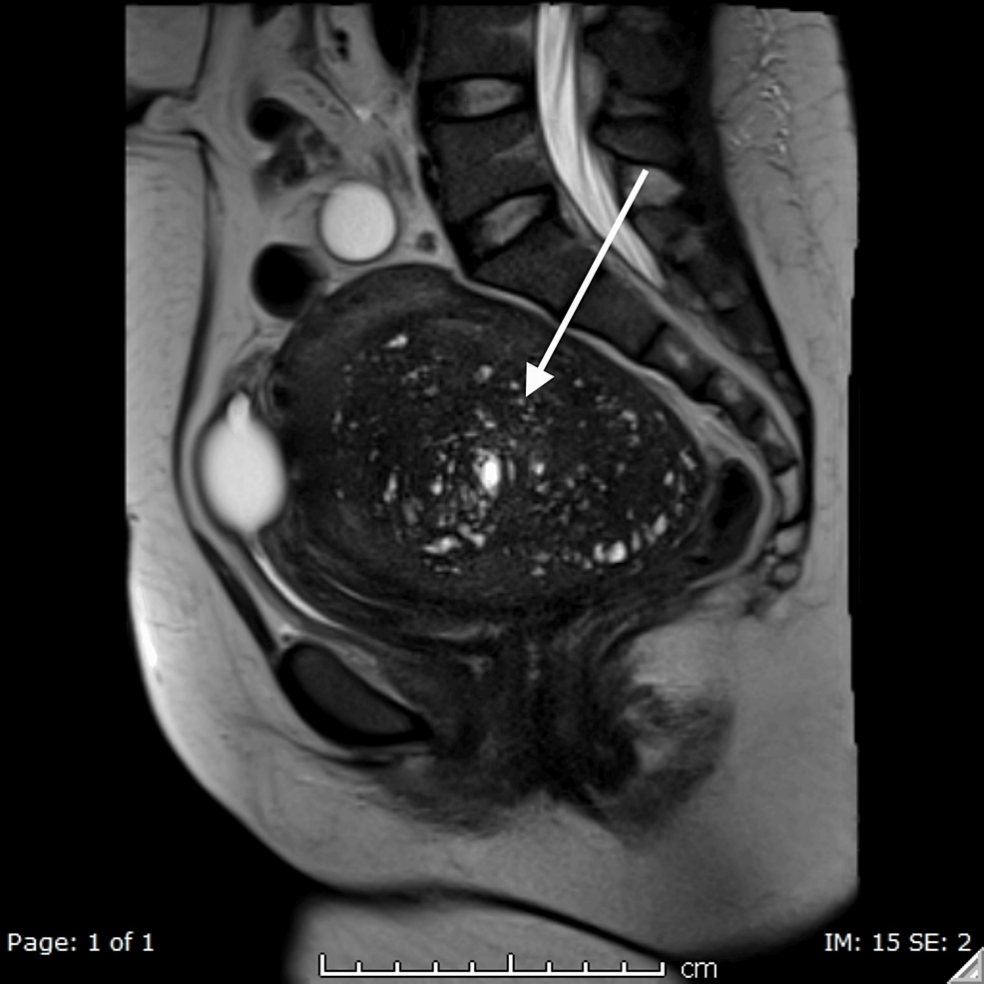
Sagittal T2-weighted images showing adenomyosis with glandular, cystic, and solid components

**Figure 2:**
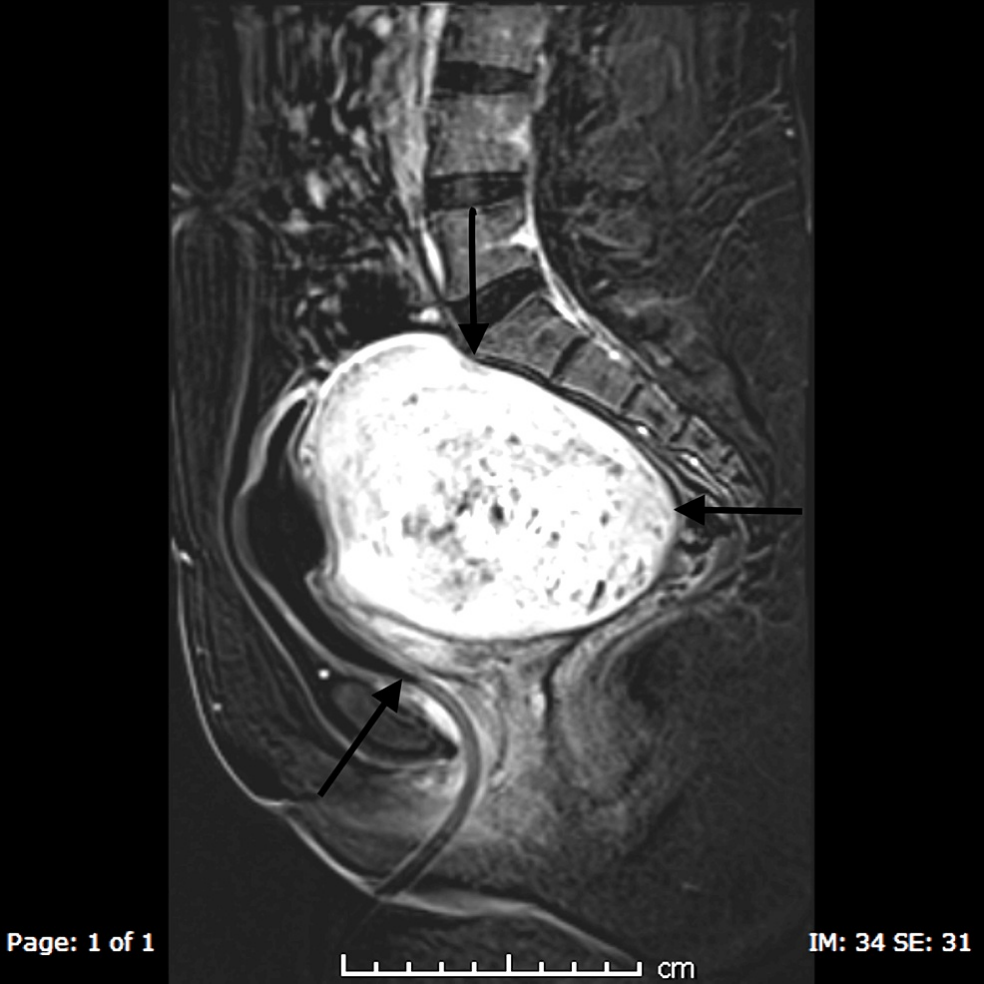
Post-contrast image showing enhancement of adenomyosis Anteriorly, the bladder can be seen being compressed by adenomyosis

Her blood test showed a hemoglobin level of 7 g/dL. Beta hCG was not elevated. Renal function, urinalysis, and urine microbiology test were normal. A transvaginal ultrasound scan of the pelvis revealed an enlarged uterus of 12.3 x 9.7 cm with a heterogenous mass lesion of about 9.5 x 8.5 cm in size with internal vascularity. The mass occupied the whole pouch of Douglas and was displacing the cervix and the rest of the uterus anteriorly. There was hydrosalpinx on the left side measuring 7.9 x 4 cm. An MRI was recommended to obtain a clearer anatomical depiction. MRI pelvis with contrast demonstrated a retroverted bulky uterus. There was a well-defined mass lesion arising from the posterior and left side of the uterus (Figure 1). The maximum size of the lesion was 9.3 cm superior inferiorly, 7.3 cm transversely, and 8.1 cm mediolaterally. The sagittal T2-weighted image showed a thickened posterior junctional line with cystic changes suggestive of adenomyosis (Figures 1-2). The entire enlarged uterus had displaced the urethra anteriorly with angulation (Figure 2).

The patient's subsequent CA 125 and CA 19-9 were found to be elevated [CA 125 level: 690 U/ml (normal: <35 U/ml; CA 19-9: 49 U/ml (normal 27U/ml)]. 

Treatment

After initial resuscitation and ensuring that the patient was stable, definitive management options were discussed, including GnRH injections vs. total abdominal hysterectomy and bilateral salpingectomy with ovary conservation. Given the patient’s concern about the recurrence of symptoms based on the size and anatomical pressure leading to incarceration and further possibility of neurological complications, she was counseled about choosing a surgical option. After a detailed discussion about the procedure and possible complications, the patient consented to a total abdominal hysterectomy since she was not planning to have any future pregnancies. Anemia was corrected preoperatively with a transfusion of two units of packed red blood cells in anticipation of possible intraoperative bleeding.

Total abdominal hysterectomy with bilateral salpingectomy was performed via Pfannenstiel incision. The uterus was found to be approximately 14 x 14 cm in size, retroverted, and incarcerated in the cul de sac. There was restricted mobility due to adhesions to the adjacent small bowel and left pelvic side wall. Hydrosalpinx was present on the left side, which was also adherent to the adjacent small bowel and pelvic side wall. To prevent ureteric injury, a double J stent was inserted in the left ureter by the urologist. Ovaries were macroscopically normal and were conserved. 

The patient's post-operative recovery was uneventful. A successful trial of the removal of the urinary catheter was carried out on the second day after surgery. A bedside urinary bladder scan revealed minimal post-void urine. She was discharged on the third day after surgery. The histopathology report revealed extensive adenomyosis without any feature of leiomyoma. There was hydrosalpinx of the left tube. One month after the surgery, the left ureteric stent was removed. She was followed up at one and three months after surgery and was found to be doing well. Her symptoms of frequent urination and the sense of incomplete emptying of the bladder were completely resolved.

## Discussion

The reported yearly incidence of urinary retention due to bladder outflow obstruction in women of reproductive age is about seven per 100,000 [7]. It is understood that gynecological conditions usually cause bladder outflow obstruction due to external compression on the bladder neck or urethra. The conditions causing such compression include prolapse of the pelvic organ, huge cystocele, and incarceration of retroverted uterine myoma or gravid uterus [8]. There have been reported cases of uterine myoma causing such obstruction, but, despite the similarities, adenomyosis of the uterus causing such obstruction is rare. The pathophysiological mechanisms underlying the development of urinary retention in cases of adenomyosis are unclear and may be slightly different. It is postulated that in such cases, the infiltration of endometrial tissue into the myometrium may result in local inflammation and distortion of nearby structures, leading to urinary dysfunction.

Our patient complained of frequent urination and a sense of incomplete bladder emptying which preceded full retention. These initial symptoms could have been due to bladder irritation. The subsequent growth of the uterine adenomyosis, however, resulted in its impaction against the sacrum posteriorly, leading to the antero-inferior displacement of the cervix against the pubic symphysis (Figure 1). CA 125 was found to be raised in our patient during preoperative workup, which may raise concerns for ovarian epithelial carcinoma; however, benign conditions such as fibroids, endometriosis, and adenomyosis are also possible causes as confirmed on histopathology [9].

Acute urinary retention is an emergency condition requiring immediate management. It is associated with risks such as obstructive uropathy, bladder rupture, bladder dysfunction, uterine infections, urinary tract infections, rectal gangrene, and bilateral hydronephrosis [10,11]. The first step in its management is emergent bladder decompression with the insertion of Foley's catheter. A detailed gynecological history and physical examination are warranted in such cases. Radiological investigations such as pelvic ultrasound and MRI are invaluable in diagnosing underlying conditions [12,13].

Positional maneuvers such as putting the patient in knee-chest or all-four position may be considered. This aids by correcting the retroverted uterus. An attempt at a manual reduction while performing a vaginal examination may prove to be successful. The technique involves the application of gentle but firm cephalad digital pressure, trying to push the prolapsed uterus toward the posterior cul de sac. The operative reduction should be considered when conservative measures fail.

## Conclusions

While adenomyosis is a rare entity, this case report underscores the significance of considering it as a possible etiology of urinary retention secondary to uterine incarceration in females of reproductive age. Timely recognition and appropriate management are vital for averting potential complications. Further research is warranted to elucidate the mechanisms underlying urinary symptoms in adenomyosis, with irritation and incarceration leading to outlet obstruction playing a possible role. Although neurological compression causing similar symptoms has been reported in cases of uterine leiomyoma, adenomyosis should also be considered when encountering females with such presentations, necessitating the exploration of optimal treatment strategies.
